# Statistical filtering for NMR based structure generation

**DOI:** 10.1186/1758-2946-3-31

**Published:** 2011-08-11

**Authors:** Jochen Junker

**Affiliations:** 1 Fundação Oswaldo Cruz - CDTS, Rio de Janeiro - RJ, Brazil

## Abstract

The constitutional assignment of natural products by NMR spectroscopy is usually based on 2D NMR experiments like COSY, HSQC, and HMBC. The difficulty of a structure elucidation problem depends more on the type of the investigated molecule than on its size. Saturated compounds can usually be assigned unambiguously by hand using only COSY and ^13^C-HMBC data, whereas condensed heterocycles are problematic due to their lack of protons that could show interatomic connectivities. Different computer programs were developed to aid in the structural assignment process, one of them COCON. In the case of unsaturated and substituted molecules structure generators frequently will generate a very large number of possible solutions. This article presents a "statistical filter" for the reduction of the number of results. The filter works by generating 3D conformations using smi23d, a simple MD approach. All molecules for which the generation of constitutional restraints failed were eliminated from the result set. Some structural elements removed by the statistical filter were analyzed and checked against Beilstein. The automatic removal of molecules for which no MD parameter set could be created was included into WEBCOCON. The effect of this filter varies in dependence of the NMR data set used, but in no case the correct constitution was removed from the resulting set.

## Findings

Nuclear Magnetic Resonance is the most common tool used for the structure elucidation of new compounds. The used 2D NMR experiments like COSY, HSQC, and ^13^C-HMBC deliver correlation information between atoms that can be translated into connectivity information. Out of these, correlation information from COSY and HSQC experiments can be transcribed directly into connectivity between atoms. But the ^13^C-HMBC correlations need more attention because of their ambiguity and complexity. Hence the difficulty of the structure elucidation problem depends more on the type of the investigated molecule than on its size. Saturated compounds can usually be assigned unambiguously using mainly COSY and some ^13^C-HMBC data, whereas condensed heterocycles are problematic due to their lack of protons that could show interatomic connectivities. This ambiguity has driven the development of different software packages to aid in the interpretation of the ^13^C-HMBC correlation data [1-19] as much as the development of additional correlation experiments [20,21].

When the observed connectivity information is used as input for the structure generation program COCON[3,22-24] it will create all compatible constitutional assignments. In the case of unsaturated molecules COCON will usually generate a very large number of possible solutions. Since the solutions will then have to be checked manually for their chemical feasibility and sense, Different efforts have been made to reduce the number solutions. Among others, ranking of the constitutional assignments by chemical shift deviation and/or substructural elements have been tested [25,26] integrated to COCON runs. Unfortunately, the described software could not be made available for the online version of COCON (WEBCOCON at http://cocon.nmr.de), since it uses data protected by Intellectual Property. A different way of handling the result set had to be chosen, and the statistical filter was implemented.

The idea behind the filter is, to compare the suggested constitutions against existing molecules, like the ones contained in the PubChem (PubChem can be found at http://pubchem.ncbi.nlm.nih.gov/) database. For each COCON-suggested constitution all 1 sphere elements of the constitutions are checked for corresponding elements in PubChem. This comparison is done indirectly, by generating molecular dynamics parameters in smi23d. The software smi23d (smi23d is available under the Apache 2.0 license and can be downloaded from http://www.chembiogrid.org/cheminfo/smi23d/) has been used to generate 3D coordinates for almost 13M compounds contained in PubChem (The corresponding 3D coordinates generated by smi23d can be found at http://www.chembiogrid.org/cheminfo/p3d/; the error observed is ~ 0.4% (= 53.000) false negatives for 13M compounds) and succeeded on generating coordinates for 99.6% of the molecules contained in the Database. The filtering application actually uses smi23d to generate 3D coordinates for all constitutional assignments generated by COCON and eliminates those for which smi23d fails because of lacking parameters. Since smi23d has successfully been used on so many well known compounds, this means that the structural element for which parameters were missing has hardly ever been observed and therefore might not exist in natural products. Due to the nature of the filter, no ranking of the remaining constitutions is carried out and further methods might be necessary to improve the results. All calculations were run on the publicly available WEBCOCON server, using the input files provided there as examples. Calculation times varied from several minutes to two hours for **1 **and **2**. For **3 **the longest running time was 3 days for the generation of the 523.668 constitutional assignments using COSY, ^13^C-HMBC correlations and open atom types. A webpage allowing direct access to the results of the structure generator runs presented here has been set up on the WEBCOCON server http://cocon.nmr.de/StatisticalFilter/ (The results are also mirrored at http://science.jotjot.net/StatisticalFilter/).

Ascomycin is a well known ethyl derivative of Tacrolimus, it serves as example of a large natural product, featuring 43 Carbon atoms. Using experimental NMR correlation data (COSY and ^13^C-HMBC correlations) together with fixed atom types, COCON generates only one solution, independent of the statistical filter. Additionally the filter showed no effect on the number of constitutional assignments generated, when no atom types were defined, in which case a total of 100 different constitutions were proposed.

**Figure 1:**
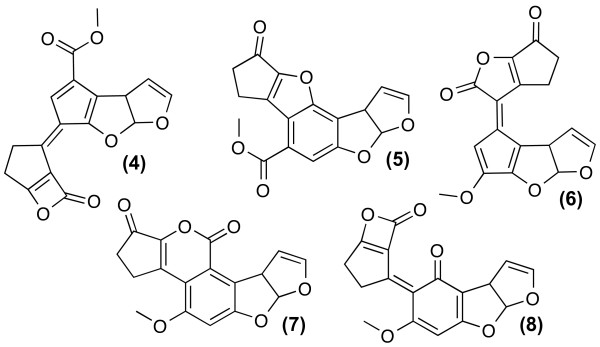
**The constitutions 4-8 shown here are excluded by the statistical filter**. Each constitution appears with two Different ^13^C chemical shift assignments in the solution set.

**Figure 2:**
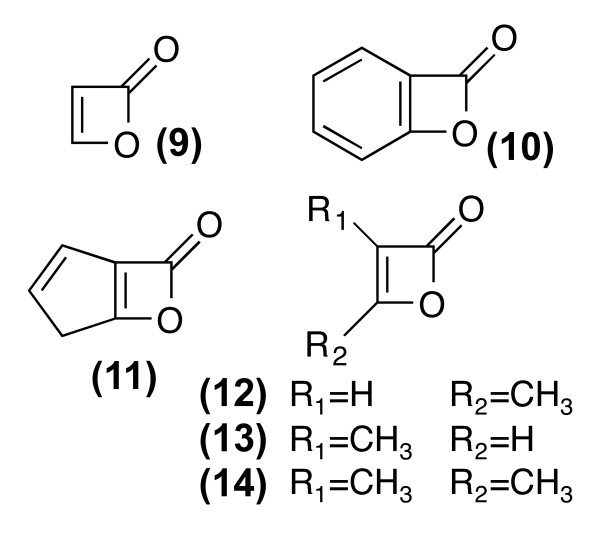
**Basic variations of the structural element Oxet-2-one that is excluded by the statistical filter, as found in 85 hits from PubChem**.

The results change with the second example molecule, Aflatoxin B1 with 17 Carbon atoms. Using COSY and ^13^C-HMBC data alone, COCON generates 970 structures. This drops to 539 results after filtering, a reduction by 45%. When the atom types are predefined COCON still generates 68 constitutional assignments, that are reduced to 58 after filtering, a reduction by 15%. The ten excluded constitutions (see Figure 1) all contain oxet-2-one as structural element, that can be found in 6 basic variations in 85 compounds in PubChem (see Figure 2). Until now, no natural product has been described with this substructure. The numbers of results for the different COCON runs for **1 **and **2 **are summarized in table 1. Oroidin **3 **has been frequently used for the demonstration of COCON. It' s relatively low number of protons and therefore small number of experimentally available COSY and ^13^C-HMBC correlations lead to a total of 523.668 possible constitutional assignments, out of which only 1904 belong to the correct atom type combination. After the statistical filtering there are still 252.566 respectively 1486 suggestions left. In this case the reliable structure elucidation by NMR needs ^15^N-HMBC or 1,1-ADEQUATE correlations. For calculations with open atom types, only when using both kinds of correlation information and filtering, a reasonable amount of 275 suggested constitutions is generated.

**Table 1:** Number of constitutional assignments suggested for 1 and 2.

	open atom types		fixed atom types	
		
	no filter	after filter	no filter	after filter
**1**	100	100	1	1
**2**	970	539	68	58

**Figure 3:**
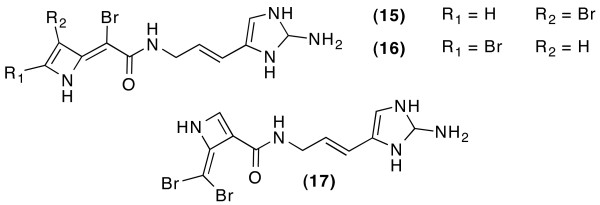
**Constitutional assignments excluded by the statistical filter when the structure generator runs with COSY, HMBC, ^15^N-HMBC correlation data and atom types for Oroidin**.

**Figure 4:**
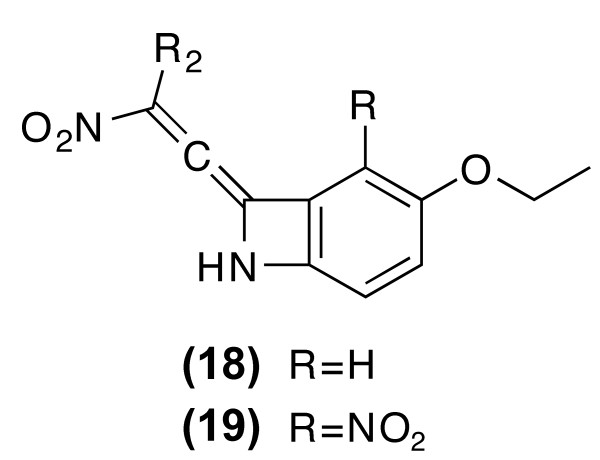
**The 14 molecules found in Beilstein containing the 1-nitro-prop-2-en-Z-ylidene substructural element all have the substitution pattern of 18 and 19**. R_2 _is either a polyaromatic or polyhalogenic substituent.

**Figure 5:**
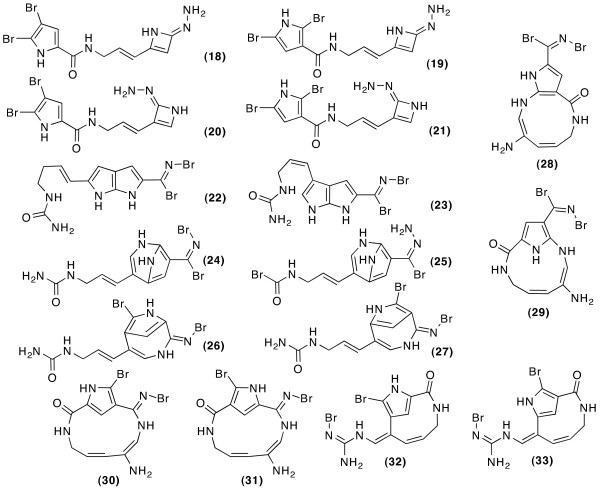
**Constitutional assignments excluded by the statistical filter when the structure generator runs with COSY, HMBC, 1,1-ADEQUATE correlation data and atom types for Oroidin**.

When the ^15^N-HMBC correlations and fixed atom types are added to the COSY and ^13^C-HMBC based calculation the statistical filter excludes only the constitutional assignments containing the 1-nitro-prop-2-en-Z-ylidene substructural element (see Figure 3). According to Beilstein, this structural element appears only 14 times, always in conjunction with an aromatic ring, as depicted in Figure 4. When 1,1-ADEQUATE correlations are added instead, and atom types are fixed, the filter excludes 16 constitutions, shown in Figure 5. All resulting numbers of constitutional assignments for the Different combinations of correlation data are summarized in table 2.

**Table 2:** Number of constitutional assignments suggested for 3 depending on the type of correlation information used.

	open atom types		fixed atom types	
		
	no filter	after filter	no filter	after filter
**CH**	523,668	252,566	1,904	1,486
**CHA**	39,025	13,473	86	70
**CHN**	716	275	17	14
**CHAN**	81	17	2	2

The correlations are encoded as: **C **= COSY, **H **= ^13^C-HMBC, **N **= ^15^N-HMBC and **A **= 1,1-ADEQUATE.

**Figure 6:**
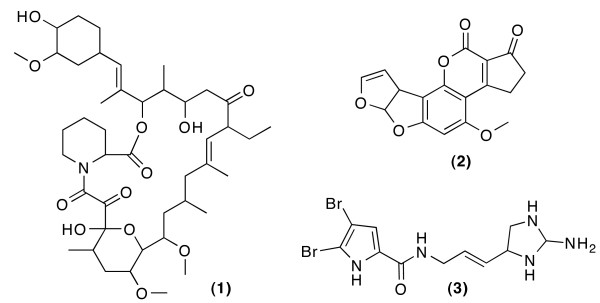
**Ascomycin 1, Aflatoxcin B1 2 and Oroidin 3 are used to evaluate the statistical filter**.

The results from tables 1 and 2 show that the filter excludes more constitutional assignments when the atom types are undefined (45% - 65%) then when the atom types are defined (~ 20%). In neither case the correct constitutions were excluded, and in the case of Ascomycin the filter did not exclude any constitutional assignment. The calculation time increases depending on the number of possible constitutional assignments, as smi23d runs about 0.5 s per structure. This explains the observed 3 days for the generation of the 523,668 constitutional assignments for Oroidin using COSY, ^13^C-HMBC correlations and open atom types, COCON itself only needed about 21 minutes. It would take considerably more time to sort out the 271,102 excluded constitutional assignments manually. Whilst nobody will manually examine the 523,668 resp. 252,566 constitutional assignments obtained for Oroidin (see table 2), looking at a mere 275 constitutional assignments instead of 716 is a considerable improvement. When looking at the excluded constitutions, and checking for the common structural elements, it turns out that they are not stable or do not exist in PubChem. Run times for the filter could be cut down in the future by restricting the MD run to just the generation of the parameters, but this would need changing the existing smi23d software package, and throwing away the possibility of improved visualization of the results with the 3D structures. The new statistical filtering presented here has already been made available in WEBCOCON, optionally only the filtered results of runs of the structure generator may be exhibited. Statistical filtering has been applied to the COCON runs for the structure elucidation of Ascomycin **1**, Aflatoxcin B1 **2 **and Oroidin **3 **(Figure 6), example molecules that have already been used on other occasions. The molecules are available as examples on the WEBCOCON server, together with the results presented here.

## Availability

The WEBCOCON server is freely accessible via http://cocon.nmr.de.

## Competing interests

The author declares that they have no competing interests.

## Authors' contributions

JJ maintains the WEBCOCON software, has implemented the changes and has run all the calculations shown.
